# Effect of Polyethylene Glycol on the NiO Photocathode

**DOI:** 10.1186/s11671-017-2267-6

**Published:** 2017-08-17

**Authors:** Shengjun Li, Zeng Chen, Wenping Kong, Xiyang Jia, Junhao Cai, Shaokang Dong

**Affiliations:** Key Laboratory of Photovoltaic Materials of Henan Province and School of Physics and Electronic, Henan University, Kaifeng, 475001 People’s Republic of China

**Keywords:** Photocathode, Nickel oxide, Polyethylene glycol, Quantum dots, p–n-Type tandem solar cells

## Abstract

In this study, a uniform nanoporous NiO film, with a thickness of up to 2.6 μm, was prepared using polyethylene glycol (PEG). The addition of PEG significantly decreased the cracks in the NiO film and prevented the peeling of the NiO film from a fluorine-doped tin oxide substrate. The NiO cathode was prepared using CdSeS quantum dots (QDs) as the sensitizer, with an optimized photoelectric conversion of 0.80%. The optimized QD-sensitized NiO films were first assembled with the TiO_2_ anode to prepared QD-sensitized p–n-type tandem solar cells. The open circuit voltage was greater than that obtained using the separated NiO cathode or TiO_2_ anode.

## Background

Solar energy demonstrates potential as the primary source of energy in the future because of its cleanliness, high power, rapid processing, and wide availability [1, 2]. Since the development of solar cells in the past 30 years, sensitized solar cells have become efficient devices for the utilization of solar energy. However, these studies focus on n-type solar cells, which are based on a sensitized n-type photoanode, e.g., TiO_2_, ZnO, and SnO_2_ [2–6]. The short-circuit current density was greater than 15 mA cm^−2^, and the photoelectric conversion efficiency was approximately 13% [5]. He et al. have reported the use of p–n-type tandem dye-sensitized solar cells (DSSCs) [7], which possibly afford a higher open-circuit voltage (OCV) and photoelectric conversion efficiency. Nakasa et al. have reported an OCV of 0.918 V by the combination of merocyanine NK-2684-sensitized NiO and TiO_2_ photoanode [8]. Nattestad et al. have reported a decrease in the charge recombination of the NiO photocathode by the optimization of donor–acceptor dyes and achieved an absorbed photon-to-electron conversion efficiency greater than 90% over a spectral range of 400–500 nm [9], with an open-circuit voltage of 1079 mV. This value is the highest value reported thus far for p–n-type tandem DSSCs.

To obtain higher photocurrents comparable to n-type photoanodes, one way is preparing new p-type cathode [10, 11]. Another way is to prepare thick mesoporous photocathodes which are preferable for adsorbing a large amount of dye molecules. Some attempts have been made to improve the thickness of NiO films; however, the photocurrent density generated is still an order of magnitude less than that observed for n-type DSSCs, and thick films often suffer from poor mechanical stability. Wu et al. have prepared NiO films by the hydrothermal method and improved their properties by the optimization of the film thickness and specific surface area [12]. Qu et al. have fabricated layered NiO films from wrinkled porous NiO nanosheets and reported significantly improved photocurrent and photovoltage [13]. Zhang et al. have improved the photovoltage by the application of highly crystalline NiO [14]. Powar et al. have obtained a high photocurrent of 7.0 mA cm^−2^ using nanostructured NiO micro-balls as active materials for the photocathode [15]. Sumikura et al. have prepared nanoporous NiO films by the hydrolysis of NiCl_2_ in a water/ethanol mixed solution using a series of polyethyleneoxide–polypropyleneoxide–polyethyleneoxide (PEO–PPO–PEO) triblock copolymers as the template [16]. They investigated the effects of the PEO–PPO–PEO template in detail. Li et al. have adopted the preparation method used by Sumikura et al. and prepared thick NiO films by a two-step doctor blading method [17]. They obtained a record incident photon-to-current efficiency (IPCE) of 64% and a short circuit current (*J*_*SC*_) of 5.48 mA cm^−2^. However, the photoelectric conversion efficiency of the p-type NiO electrode is maintained between 0.02 and 0.3% using different dyes. In this experiment, precursor solutions of NiO were prepared using F108 (polyethyleneoxide–polypropyleneoxide–polyethyleneoxide (PEO–PPO–PEO) triblock co-polymers, MW: ca. 14,600) as the template following Sumikura et al.’s method. Polyethylene glycol (PEG; MW: ca. 20,000) was added into the precursor solution, and its effects on the NiO film were investigated in detail. Finally, a p–n-type quantum dot (QD)-sensitized tandem solar cells were also assembled.

## Experimental

A precursor solution of NiO was prepared according to a previously reported method [17]. First, anhydrous NiCl_2_ (1 g) and F108 (1 g) were dissolved in a mixture of deionized water (3 g) and ethanol (6 g). Second, the solution was left to rest for 3 days. Third, a specific content of polyethylene glycol (MW of 20,000) was added into the NiO precursor solution. Next, the mixture was stirred for 4 h and centrifuged at 8000 rad/min. The PEG content was controlled at 0.03, 0.075, 0.15, and 0.3 g. The above solution was deposited on a fluorine-doped tin oxide (FTO) glass substrate by the doctor blading method and dried at room temperature. The films were sintered at 400 °C for 30 min under air. CdSeS QDs were prepared by hot-injection synthesis according to previous experiments reported by our group [18]. The prepared NiO films were sensitized by CdSeS QDs by the electrophoretic method using a mixed acetonitrile/toluene (1:2.5 *v*/*v*) solution by applying a DC of 50 V for a specific time. TiO_2_ films were co-sensitized with CdS/CdSe using the conventional successive ionic layer adsorption and reaction (SILAR) method [19]. QD-sensitized TiO_2_ films were used as the anode instead of CuS to assemble p–n-type QD-sensitized solar cells.

The morphology of the NiO films was examined using a JSM-7001F field-emission scanning electron microscope (FE-SEM). Photocurrent density–voltage (*J*–*V*) characteristics were measured using a Keithley 2440 source meter under AM 1.5G illumination from a Newport Oriel solar simulator with an intensity of 1 Sun.

## Results and Discussion

**Fig. 1:**
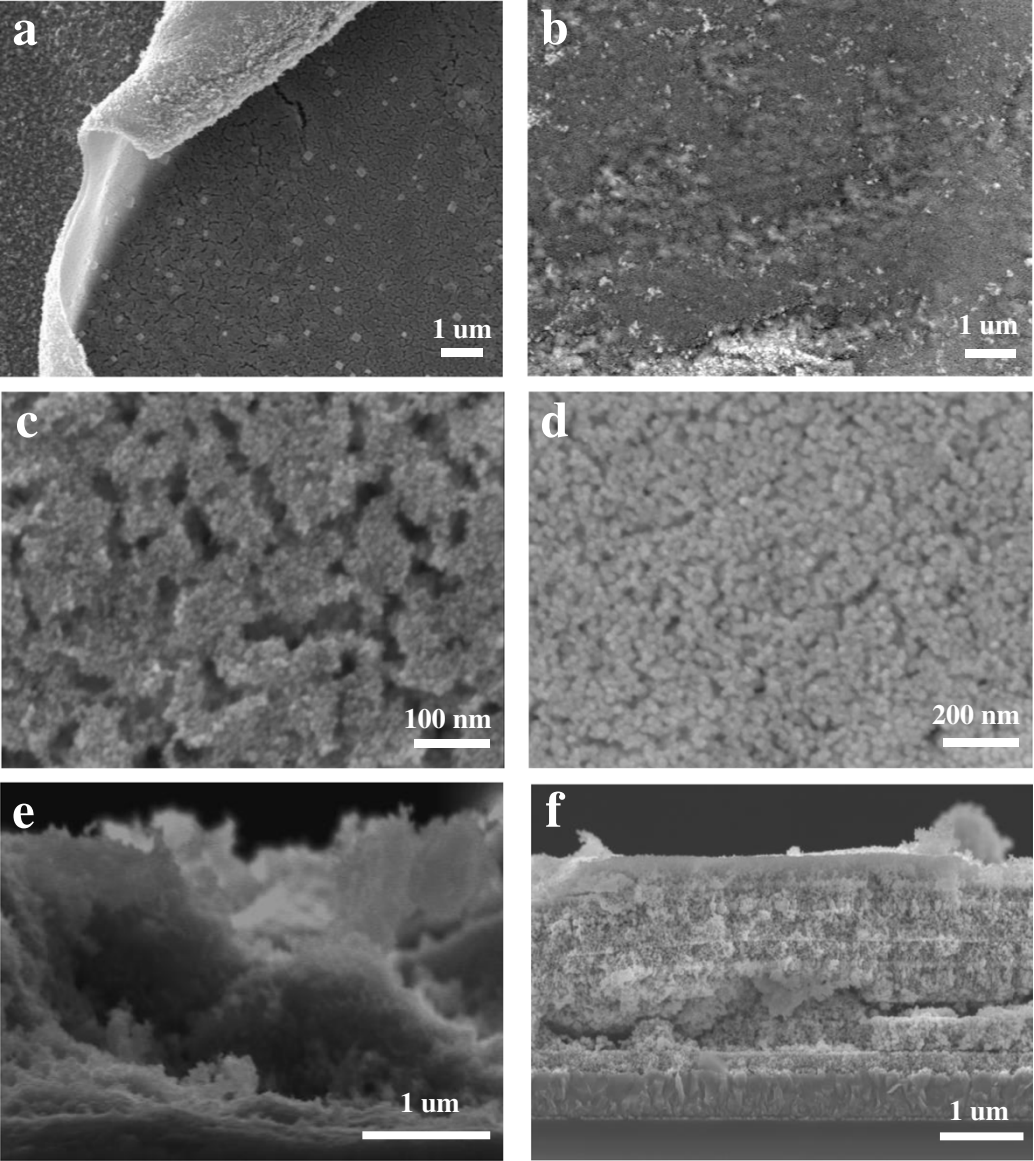
SEM micrographs of the NiO films: **a**, **c**, and **e** were fabricated from the precursor solution without polyethylene glycol. **b**, **d**, and **f** were fabricated from the precursor solution with polyethylene glycol

The NiO film was prepared by the doctor blading method. The film would peel off in case of the NiO precursor solution without PEG when the blading time was greater than four times. Figure 1a, c, e shows the surface and cross morphology of the NiO films bladed four times. The NiO films, which exhibited several micro-ravines, curled up from the FTO substrate. Figure 1b, d, f shows the surface and cross section of the NiO films prepared using PEG. The films were bladed seven times. Almost no cracks in the NiO films were observed. The particle size was less than that of the NiO film prepared without PEG. In addition, clear changes were observed in the cross sections of these two NiO films prepared with or without PEG. The NiO film prepared using the NiO precursor solution without PEG was apparently composed of nanosheets. In fact, these nanosheets should appear as curled NiO films, which could peel off from the FTO substrate. However, the NiO films prepared using the NiO precursor solution with PEG comprised several layers, with each NiO film layer being bound to the other layers. There were no obvious cracks between different layers, with a thickness of approximately 2.6 μm. PEG might have two effects in the formation process of NiO film. One was that PEG might improve the joint between these NiO particles and decrease the appearing of cracks at the drying process after the NiO gels were bladed on the FTO substrate. Meanwhile, PEG can be used as structure-directing agent. The addition of PEG can improve the specific surface area and pore volume of NiO film.

**Fig. 2:**
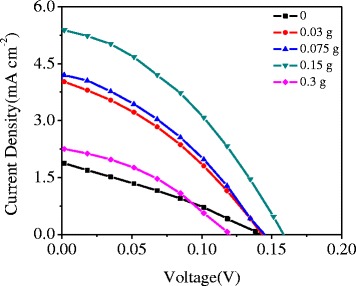
Current density–voltage characteristic curves of the NiO photocathodes with different content of PEG in the precursor solution

The prepared NiO films with two layers were sensitized with CdSeS QDs by electrophoretic deposition. The photocurrent–voltage (*J*–*V*) curves were recorded under an intensity of 1 Sun using the Newport Oriel solar simulator as the light source. Figure 2 shows the *J–V* curves thus obtained. As can be observed from Fig. 2, with the addition of 0 to 0.15 g PEG, the conversion efficiency was significantly improved from 0.08 to 0.32%. The OCV, *J*_*SC*_, and fill factor (FF) for the best NiO photocathode was 0.158 V, 4.40 mA cm^−2^, and 0.46, respectively. The property would sharply decay with the change in the PEG content from 0.15 to 0.3 g. Hence, the concentration of PEG in the NiO precursor solution significantly affected the property of the NiO cathode.

**Fig. 3:**
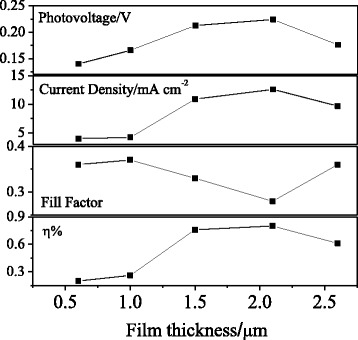
Effect of film thickness on the photovoltaic characteristics of the NiO photocathodes

The effects of the NiO film thickness were also investigated. In this experiment, the content of PEG was fixed at 0.15 g. Figure 3 shows the curves of photoelectric properties. With the increase in the film thickness from 0.6 to 2.1 μm, the OCV and *J*_SC_ increased. Both these factors tended to decay with the further increase in the film thickness. The FF exhibited almost no changes with the increase of film thickness. These weak changes might be related to the increase in the photocurrent density. As a result, the photoelectric conversion efficiency increased with the initial thickening of the NiO film. Weak changes were observed for a film thickness greater than 1.5 μm, related to the low hole transport rate and short hole lifetime [20].

**Fig. 4:**
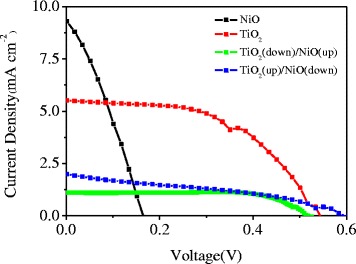
Current density–voltage characteristic curves of p–n-type quantum dot-sensitized p–n-type tandem solar cells

The prepared NiO cathode was assembled together with the TiO_2_ anode to prepare QD-sensitized p–n-type tandem solar cells. Figure 4 shows the *J–V* curves of the NiO cathode and the TiO_2_ anode, as well as the tandem TiO_2_(down)/NiO(up) and TiO_2_(up)/NiO(down) solar cells. The p–n-type tandem solar cells with TiO_2_(down)/NiO(up) configuration exhibited significantly enhanced OCV as compared to the separated NiO cathode or TiO_2_ anode. The photoelectric conversion efficiency was 0.43%, with an OCV of 0.594 V, *J*_SC_ of 2.0 mA cm^−2^, and an *FF* of 0.36. This is the first study about the QD-sensitized p–n-type tandem solar cells. However, the *J*_SC_ of the tandem solar cells was significantly less than those of the NiO cathode and TiO_2_ anode. In addition, the photoelectric conversion efficiency was less than those of the NiO cathode and TiO_2_ anode. In the future, more studies should be conducted to enhance the high performance of QD-sensitized p–n-type tandem solar cells.

## Conclusion

Polyethylene glycol (PEG) was used to prepare NiO films. The addition of PEG significantly decreased the cracks in the NiO films. A uniform 2.6-μm-thick nanoporous NiO film was prepared. The optimized photoelectric conversion efficiency was 0.80%. The optimized quantum dot-sensitized NiO film was first assembled with the TiO_2_ anode to prepared QD-sensitized p–n-type tandem solar cells. The open-circuit voltage (OCV) was greater than that exhibited by the separated NiO cathode or TiO_2_ anode. The TiO_2_(down)/NiO(up) tandem solar cells afforded a total photoelectric conversion of 0.43%, with an OCV, short circuit current density, and fill factor of 0.594 V, 2.0 mA cm^−2^, and 0.36, respectively.
